# Advances in wearable technology and applications in physical medicine and rehabilitation

**DOI:** 10.1186/1743-0003-2-2

**Published:** 2005-02-25

**Authors:** Paolo Bonato

**Affiliations:** 1 Department of Physical Medicine and Rehabilitation, Harvard Medical School and The Harvard-MIT Division of Health Sciences and Technology, Spaulding Rehabilitation Hospital, 125 Nashua Street, Boston MA 02114, USA

## Abstract

The development of miniature sensors that can be unobtrusively attached to the body or can be part of clothing items, such as sensing elements embedded in the fabric of garments, have opened countless possibilities of monitoring patients in the field over extended periods of time. This is of particular relevance to the practice of physical medicine and rehabilitation. Wearable technology addresses a major question in the management of patients undergoing rehabilitation, i.e. have clinical interventions a significant impact on the real life of patients? Wearable technology allows clinicians to gather data where it matters the most to answer this question, i.e. the home and community settings. Direct observations concerning the impact of clinical interventions on mobility, level of independence, and quality of life can be performed by means of wearable systems. Researchers have focused on three main areas of work to develop tools of clinical interest: 1)the design and implementation of sensors that are minimally obtrusive and reliably record movement or physiological signals, 2)the development of systems that unobtrusively gather data from multiple wearable sensors and deliver this information to clinicians in the way that is most appropriate for each application, and 3)the design and implementation of algorithms to extract clinically relevant information from data recorded using wearable technology. Journal of NeuroEngineering and Rehabilitation has devoted a series of articles to this topic with the objective of offering a description of the state of the art in this research field and pointing to emerging applications that are relevant to the clinical practice in physical medicine and rehabilitation.

## The potential impact of wearable technology on physical medicine and rehabilitation

Understanding the impact of clinical interventions on the real life of individuals is an essential component of physical medicine and rehabilitation. While assessments performed in the clinical setting have value, it is difficult to perform thorough, costly evaluations of impairment and functional limitation within the time constraints and limited resources available in outpatient units of rehabilitation hospitals. Furthermore, it is often questioned whether assessments performed in the clinical setting are truly representative of how a given clinical intervention affects the real life of patients. While this observation has fostered a great deal of interest for the development and validation of outcome measures that largely rely on the use of questionnaires [1], researchers and clinicians have looked at recent advances in wearable technology intrigued by the possibility offered by this technology of gathering sensor data in the field [2,3]. Likely to be complementary to outcome measures, the use of wearable systems in the clinical management of individuals undergoing rehabilitation is very attractive because it provides the opportunity of recording quantitative data in the settings that matter the most, i.e. the home and the community.

A number of clinical applications of wearable systems in physical medicine and rehabilitation emerged in the past few years. They range from simple monitoring of daily activities, for the purpose of assessing mobility and level of independence in individuals, to integrating miniature sensors to enhance the function of devices utilized by patients to perform motor tasks that they would be otherwise unable to accomplish.

Monitoring functional motor activities was one of the first goals of research teams interested in clinical applications of wearable technology. The focus was initially on using accelerometers [4-8] or a combination of accelerometers and electromyographic sensors [9] to capture movement and muscle activity patterns associated with a given set of functional motor tasks. The set of tasks to be identified varied according to the clinical application. Their study was combined with monitoring systemic responses when the clinical assessment required combining motor activities and cardio-respiratory data such as in the clinical management of patients with chronic obstructive pulmonary disease [10].

A level of complexity was added when researchers started investigating motor disorders and the possibility of utilizing wearable technology to assess the effect of clinical interventions on the quality of movement observed while patients performed functional tasks. Two applications worth mentioning are the one to assess symptoms and motor complications in patients with Parkinson's disease [11-14] and the study of motor recovery in post-stroke individuals [15-17]. This shift from identifying functional motor activities to studying motor patterns associated with motor disorders generated significant interest for more complex ways to monitor movement, i.e. utilizing not only accelerometers but also gyroscopes and magnetometers or inclinometers. The combination of multiple sensors allows one to estimate the kinematics of movement [18-21] with a reliability that cannot be obtained by solely relying on accelerometers [22].

Finally, recent studies have been focused on integrating wearable, miniature sensor technology with orthoses, prostheses, and mobility assistive devices. Sensor technology is particularly appealing in these applications because it allows implementing closed-loop strategies that take advantage of the increased complexity and flexibility that robotics is contributing to the design of orthoses, prostheses, and mobility assistive devices. Namely, the characteristics of such devices can be constantly modified as a function of the task individuals are engaged into and environmental disturbances [23,24].

In all the emerging applications summarized above, either continuous recording of sensor data or at least monitoring over extended periods of time are necessary to design and implement an effective clinical intervention. Unobtrusive, wearable systems providing ease of data gathering and some processing capabilities are essential to achieve the objective of making the leap between the preliminary results obtained as part of the research carried on so far and the daily clinical practice of physical medicine and rehabilitation. Three areas of work are essential to achieve this objective: 1)the development of wearable sensors that unobtrusively and reliably record movement and other physiological data relevant to rehabilitation; 2)the design and implementation of systems that integrate multiple sensors, record data simultaneously from wearable sensors of different types, and relay sensor data to a remote location at the time and in the way that is most appropriate for the clinical application of interest; and 3)the development of methodologies to manipulate wearable sensor data to extract information in a clinically relevant manner to perform clinical assessments or control devices aimed at enhancing mobility in individuals with conditions that limit their level of independence. A series of papers have been assembled to provide the readership of Journal of NeuroEngineering and Rehabilitation with a description of the state of the art of the application of wearable technology in physical medicine and rehabilitation.

## Wearable sensors to measure movement and physiological signals

A first set of the papers that have been assembled for publication on Journal of NeuroEngineering and Rehabilitation on the topic of wearable technology in physical medicine and rehabilitation has the objective of describing recent advances in wearable sensor technology. Two manuscripts describe attempts by different groups of measuring angular displacements for upper and lower extremity joints by embedding conductive fibers into the fabric of undergarments. The paper by Gibbs and Asada, entitled "Wearable conductive fiber sensors for multi-axis human joint angle measurements", reports encouraging preliminary results concerning monitoring lower limb joint displacements during ambulation by utilizing such technology. The manuscript by Tognetti et al, entitled "Wearable kinesthetic system for capturing and classifying upper limb gesture in post-stroke rehabilitation", describes the design and implementation of a system similar to the one proposed by Gibbs and Asada but geared toward monitoring movements of the upper extremities. The authors also explore the application of these wearable sensors to monitoring motor recovery in post-stroke individuals. Simone and Kamper focus their contribution on unobtrusively measuring finger movements in patients undergoing rehabilitation. Their manuscript "Design considerations for a wearable monitor to measure finger posture" summarizes the authors' recent work toward developing ways to record fine motor control tasks involving manipulation of objects requiring fine motor control of the hand and fingers. This technology has immediate application in patients such as post-stroke individuals undergoing rehabilitation that targets fine motor control skills. While initial research in the area of wearable technology was aimed at combining existing, miniature sensors with special fabrics or wireless technology, recent advances in this field have been focused on the development of sensing elements that can be even more easily embedded in clothing items. An example of such effort is reported in the paper by Dunne et al entitled "Initial development and testing of a novel foam-based pressure sensor for wearable sensing". This paper summarizes positive preliminary results by the research team aimed at measuring shoulder movements, neck movements, and scapular pressure. The sensing elements can also be used to monitor respiratory rate. Devoted to monitoring systemic responses is the last of the papers focused on wearable sensors. In this manuscript, Yan et al describe a new method to reliably measure heart rate and oxygen saturation. The paper is entitled "Reduction of motion artifacts in pulse oximetry by smoothed pseudo Wigner-Ville distribution" and demonstrates how advanced processing techniques may be necessary to derive reliable data when recordings are performed in the field.

## Wearable systems to gather data unobtrusively and reliably over extended periods of time

A second area of research relevant to the application of wearable technology in physical medicine and rehabilitation concerns the integration of wearable sensors into systems. Following the seminal work by Park and Jayaraman [25], several researchers relied on conductive fabrics to deliver sensor data to a data-logger and then integrated it into a system that allowed remote access to the data. Other researchers explored the use of wireless technology as a means to relay wearable sensor data to a base station for data recording and remote access to clinically relevant information. Jovanov et al summarize recent advances by their research team toward developing body area networks in the manuscript entitled "A wireless body area network of intelligent motion sensors for computer assisted physical rehabilitation". Key points concerning the use of wireless technology in field monitoring of patients undergoing rehabilitation are the design of low-power transmission devices, the integration of multiple sensors, and the ability of providing processing capability that may reduce the amount of information to be transmitted. These issues are addressed in the above-referenced paper as well as in the manuscript by Sung et al entitled "Wearable feedback systems for rehabilitation". Sung et al describe a platform of wearable sensors recently developed by their team as well as potential applications currently under investigation.

## Clinical applications of wearable technology in physical medicine and rehabilitation

A final set of papers is focused on applications that are relevant to physical medicine and rehabilitation. Sherrill et al describe in their paper entitled "A clustering technique to assess feasibility of motor activity identification in COPD patients via analysis of wearable-sensor data" a method to design classifiers of motor activities such as walking and stair climbing. The proposed technique relies on the examination of small datasets via clustering methods. Measures are derived from clusters associated with different motor activities to evaluate whether the set of wearable sensors and features derived from the recorded data are suitable to reliably identify the motor tasks of interest. Wang and Winters put the information gathered via wearable systems into a clinical context via processing that relies on neuro-fuzzy models. Their paper entitled "A dynamic neuro-fuzzy model providing bio-state estimation and prognosis prediction for wearable intelligent assistants" presents encouraging results indicating that the proposed method can put in the correct context dynamic changes observed in post-stroke individuals undergoing rehabilitation. Wang and Kiryu in their manuscript entitled "Personal customizing exercise with a wearable measurement and control unit" summarize their results on customizing machine-based exercise routines on the basis of physiological data that are continuously gathered from individuals performing such routines. Their results demonstrate the feasibility of a closed-loop system that optimally adapts workload. Dozza et al describe a wearable system designed to reduce body sway in individuals with severe vestibular problems. Their manuscript entitled "Influence of a portable audio-feedback device on structural properties of postural sway" summarizes positive results obtained with a prototype wearable system that utilizes audio-feedback to improve balance. Finally, Mavroidis et al describe how miniature sensor technology can be used to design a new generation of smart rehabilitation devices. Three devices are described in their paper entitled "Smart portable rehabilitation devices": a passive motion elbow device, a knee brace that provides variable resistance by controlling damping via the use of an electro-rheological fluid, and a portable knee device that combines electrical stimulation and biofeedback. These devices combine sensing technology and control strategies to enhance rehabilitation.

## Conclusion

This collection of papers provides an up-to-date description of the state of the art in the field of wearable technology applied to physical medicine and rehabilitation. The field is rapidly advancing and numerous research groups have already demonstrated applications of great clinical relevance. The potential impact of this technology on the clinical practice of physical medicine and rehabilitation is remarkable. A significant shift in focus is possible thanks to wearable technology. While the main focus of clinical assessment techniques is currently on methods that are implemented in the clinical setting, wearable technology has the potential to redirect such focus on field recordings. This is expected to allow clinicians to eventually benefit from both data gathered in the home and the community settings during the performance of activities of daily living and data recorded in the clinical setting under controlled conditions. Complementarities are expected between field and clinical evaluations. Future research will surely address optimal ways to combine these two types of assessment to optimize the design of rehabilitation interventions.
